# Neuroprotective Effects of Vesatolimod in EAE: Modulating Immune Balance and Microglial Polarization

**DOI:** 10.3390/ijms26199297

**Published:** 2025-09-23

**Authors:** Xueyu Chen, Jian Zhang, Shuhua Mu

**Affiliations:** 1 School of Pharmaceutical Sciences, Health Science Center, Shenzhen University, Shenzhen 518055, China; 2 School of Psychology, Shenzhen University, Shenzhen 518060, China

**Keywords:** Vesatolimod, experimental autoimmune encephalomyelitis, neuroinflammation, microglial polarization, Nrf2 pathway, multiple sclerosis

## Abstract

Multiple sclerosis (MS) is a chronic autoimmune disease characterized by sustained neuroinflammation and demyelination within the central nervous system (CNS). Vesatolimod (VES), a selective Toll-like receptor 7 (TLR7) agonist, has demonstrated both antiviral and immunomodulatory properties; however, its potential therapeutic value in neuroinflammatory contexts remains poorly understood. In this study, we evaluated the efficacy of VES in the experimental autoimmune encephalomyelitis (EAE) model of MS and elucidated its mechanisms of action. EAE was induced in mice by immunization with myelin oligodendrocyte glycoprotein (MOG_35–55_). The therapeutic effects of VES were assessed through clinical scoring, body weight monitoring, histopathology, flow cytometry, quantitative proteomics, and Western blot analysis. Additionally, an in vitro model of lipopolysaccharide (LPS)-induced microglial activation was employed to investigate cell-autonomous mechanisms. Results showed that VES administration significantly ameliorated disease severity, reduced weight loss, and enhanced neurological function in EAE mice. Treatment with VES inhibited the differentiation of pro-inflammatory Th1 and Th17 cells while expanding regulatory T cell (Treg) populations. It also preserved blood–brain barrier (BBB) integrity, attenuated demyelination, and modulated microglial activation phenotypes within the CNS. At the molecular level, VES activated the Nrf2/HO-1 antioxidant pathway, thereby enhancing the expression of cytoprotective proteins. Proteomic profiling further revealed the downregulation of inflammation-related proteins, specifically those associated with TNF, IL-17, and NOD-like receptor signaling pathways. Collectively, these findings demonstrate that VES alleviates neuroinflammation in EAE through multimodal mechanisms—including peripheral and central immune regulation, BBB protection, and activation of endogenous antioxidant defenses—supporting its further development as a promising therapeutic candidate for MS.

## 1. Introduction

Multiple sclerosis (MS) is a leading cause of non-traumatic neurological disability among young adults, affecting an estimated 2.8 million individuals globally. The disease is driven by an aberrant immune response against central nervous system (CNS) myelin, resulting in chronic inflammation, demyelination, and progressive axonal loss [1]. Over the past two decades, disease-modifying therapies (DMTs)—such as interferon-β, fingolimod, and more recently, anti-CD20 monoclonal antibodies—have improved symptom management and reduced relapse rates [2,3]. Notably, several agents, including siponimod, ocrelizumab, and cladribine, have been specifically approved for progressive forms of MS, reflecting an evolving therapeutic landscape aimed at targeting more advanced disease stages [4,5]. Despite these advancements, treatment efficacy remains limited, particularly in progressive MS, where 30–50% of patients show minimal response [6]. This is largely due to the inability of current therapies to adequately target CNS-resident inflammation and neurodegeneration, as they primarily act on peripheral immune mechanisms [3,7]. Furthermore, long-term immunosuppression increases the risk of infections and malignancies [8]. These shortcomings underscore the need for innovative therapeutic strategies that address both peripheral and CNS-specific immune processes.

The experimental autoimmune encephalomyelitis (EAE) model, induced by immunization with myelin oligodendrocyte glycoprotein (MOG_35–55_), is widely employed in MS research due to its ability to recapitulate key disease features, including T cell activation, blood–brain barrier disruption, CNS infiltration, demyelination, and motor deficits [9,10].

Microglia, the resident immune cells of the CNS, play dual roles in MS pathogenesis. Single-cell transcriptomic studies have identified disease-associated microglia (DAM) within MS lesions, characterized by metabolic shifts and a proinflammatory phenotype [11,12]. In early disease, activated microglia release cytokines such as TNF-α and IL-1β, along with reactive oxygen species (ROS) and nitric oxide (NO), which exacerbate neuronal and oligodendrocyte injury. Acting as antigen-presenting cells, microglia also amplify T cell responses, perpetuating neuroinflammation [13,14,15]. However, microglia also exhibit neuroprotective properties. Under specific conditions, they can transition to an M2-like phenotype and secrete neurotrophic factors such as IGF-1 and BDNF, promoting remyelination and tissue repair [6,16]. This phenotypic plasticity positions microglia as a promising therapeutic target. Yet, no approved therapies currently exist that selectively modulate microglial polarization.

Vesatolimod (VES) is a selective small-molecule agonist of Toll-like receptor 7 (TLR7), originally developed for treating chronic viral infections due to its potent antiviral activity. Preclinical studies demonstrated its efficacy in woodchuck and chimpanzee models of chronic hepatitis virus infection [17], and subsequent clinical trials confirmed its safety and antiviral effects in patients with HBV, HCV, and HIV-1 (NCT04364035, NCT01591668, NCT01590641) [18,19,20,21]. However, it is important to note that several phase II trials evaluating VES for viral infections did not meet their primary efficacy endpoints. For instance, in a trial involving patients with chronic HBV, VES treatment did not result in significant HBsAg decline compared to placebo [22]. Despite these outcomes in virological settings, emerging evidence indicates that VES exerts broader immunomodulatory effects, which have prompted investigation in other therapeutic contexts. In a mouse model of enterovirus 71 (EV71) infection, VES significantly reduced proinflammatory cytokine levels, including IL-1β, IL-6, IL-12, IFN-α, and IFN-β. Mechanistic studies suggested that these effects may occur independently of canonical TLR7 signaling, involving suppression of the NF-κB and PI3K/AKT pathways instead-pointing to a broader, multitargeted mechanism of action [23]. Our previous work demonstrated that VES has therapeutic efficacy in EAE, a widely used model of MS, as reflected by improved clinical outcomes and reduced CNS inflammation [24]. Interestingly, VES treatment did not alter TLR7 expression, suggesting a TLR7-independent mode of action. The underlying mechanisms, however, remain to be elucidated.

Given the limitations of current MS therapies and the unique immunomodulatory profile of VES, this study addresses several key questions: (1) Can VES overcome the shortcomings of existing treatments by modulating both peripheral and CNS immune responses? (2) Can it influence microglial polarization to interrupt the cycle of neuroinflammation? (3) Do its TLR7-independent effects involve activation of endogenous neuroprotective pathways, such as Nrf2 signaling? To explore these possibilities, we systematically investigate VES in the EAE model, examining its effects on immune cell subsets, blood–brain barrier integrity, microglial polarization, and antioxidant responses. These findings aim to support the development of novel MS therapies that concurrently target both peripheral and central immune dysregulation.

## 2. Results

### 2.1. VES Alleviates EAE Symptoms and Enhances Neurological Recovery

**Figure 1:**
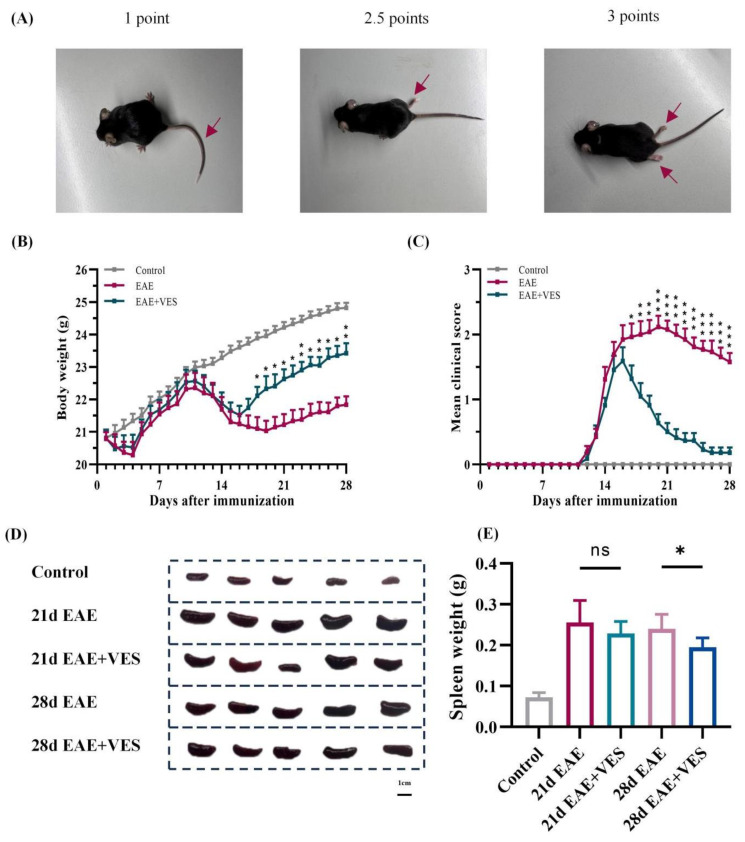
VES attenuates neurological deficits and splenomegalyin EAE mice. (**A**) Representative images of clinical symptoms; (**B**) Body weight changes over 28 days post-immunization; (**C**) Clinical scores over time (**D**) Representative spleen images; (**E**) Quantification of spleen weight. * *p* < 0.05; *n* = 25.

EAE mice developed typical clinical signs around day 12 post-immunization, including weight loss, decreased tail tone, and hind limb paralysis. The disease progressed through an acute phase (days 12–21), followed by a partial remission phase (days 21–28) (Figure 1A–C). VES treatment significantly alleviated EAE-associated metabolic dysfunction, as indicated by earlier and sustained weight recovery. By day 18 (four days post-treatment), VES-treated mice weighed significantly more than untreated EAE controls (22.11 ± 0.27 g vs. 21.10 ± 0.35 g; *p* < 0.05). This difference remained significant through day 28 (23.42 ± 0.31 g vs. 22.11 ± 0.26 g; *p* < 0.01) (Figure 1B). VES also markedly improved neurological function. Clinical scores in treated mice were significantly lower than in controls by day 17 (1.23 ± 0.23 vs. 1.92 ± 0.22; *p* < 0.05) and remained reduced throughout the acute phase. At peak severity (day 21), VES lowered clinical scores by 24.6% compared to untreated mice (1.59 ± 0.21 vs. 2.11 ± 0.17; *p* < 0.001). During remission, VES continued to promote recovery, with treated mice maintaining significantly better clinical outcomes (*p* < 0.001) (Figure 1C).

### 2.2. VES Alleviates EAE-Induced Splenomegaly

The spleen, a central peripheral immune organ, reflects systemic immune activation in autoimmune conditions. At the acute stage of EAE (day 21), mice exhibited marked splenomegaly compared to controls (0.25 ± 0.05 g vs. 0.07 ± 0.01 g; *p* < 0.01), indicating robust immune activation (Figure 1D). Although VES treatment slightly reduced spleen weight at this stage (0.23 ± 0.03 g), the difference was not significant (*p* > 0.05). By the chronic phase (day 28), however, spleen weight in VES-treated mice was significantly lower than in untreated EAE mice (0.19 ± 0.02 g vs. 0.24 ± 0.03 g; Figure 1E; *p* < 0.05), suggesting that VES suppresses sustained peripheral immune responses. These results support a potential long-term immunomodulatory role for VES in EAE.

### 2.3. VES Regulates Peripheral CD4^+^ T Cell Subsets in EAE

EAE pathogenesis is primarily driven by CD4^+^ T cell-mediated immune responses, with aberrant activation of Th1 and Th17 cells playing a central role in CNS inflammation. These subsets release proinflammatory cytokines, notably IFN-γ and IL-17A, which disrupt the BBB and trigger neuroinflammatory cascades. In contrast, regulatory T cells (Tregs) maintain immune tolerance and exert neuroprotective effects.

**Figure 2:**
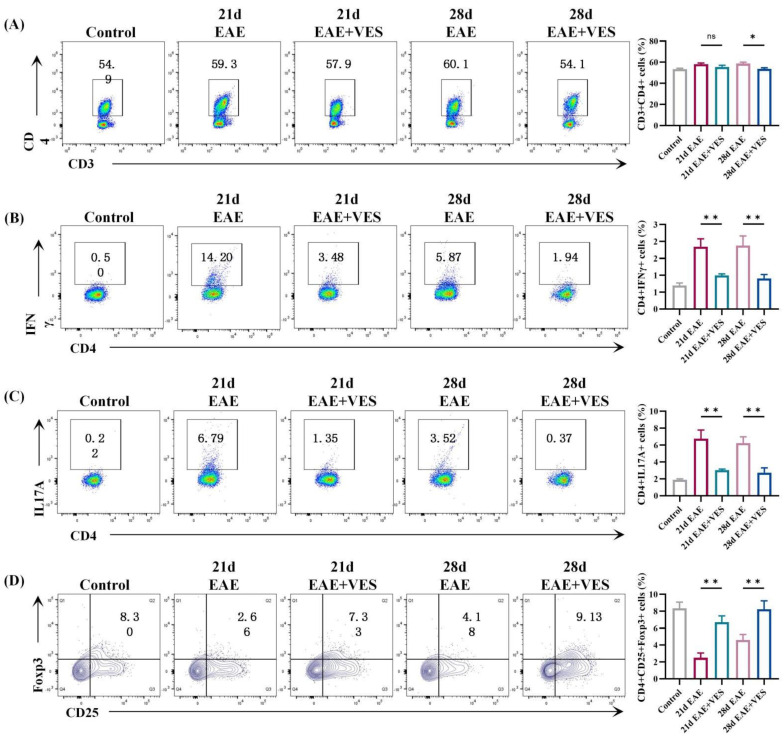
VES modulates T cell differentiation in the spleen of EAE mice. (**A**) Proportion of CD3^+^CD4^+^ cells; (**B**) Proportion of CD4^+^IFN−γ^+^ cells; (**C**) Proportion of CD4^+^IL−17A^+^ cells; (**D**) Proportion of CD4^+^CD25^+^Foxp3^+^ cells (* *p* < 0.05, ** *p* < 0.01; *n* = 5).

Flow cytometric analysis of splenic and peripheral blood lymphocytes revealed that VES effectively modulates CD4^+^ T cell subset dynamics. In the spleen (Figure 2), total CD4^+^ T cell counts were similar between EAE and VES-treated mice on day 21, but VES treatment was associated with reduced CD4^+^ T cell expansion by day 28 (*p* < 0.05). Subset analysis suggested a modulatory effect of VES, showing a marked reduction in Th1 and Th17 cells, evidenced by decreased frequencies of IFN-γ^+^ and IL-17A^+^ cells (*p* < 0.01). Concurrently, an increasing trend in the frequency of Foxp3^+^ Tregs was observed (*p* < 0.01); however, given the limited sample size (*n* = 5), this finding should be interpreted with caution and requires further validation in larger cohorts.

**Figure 3:**
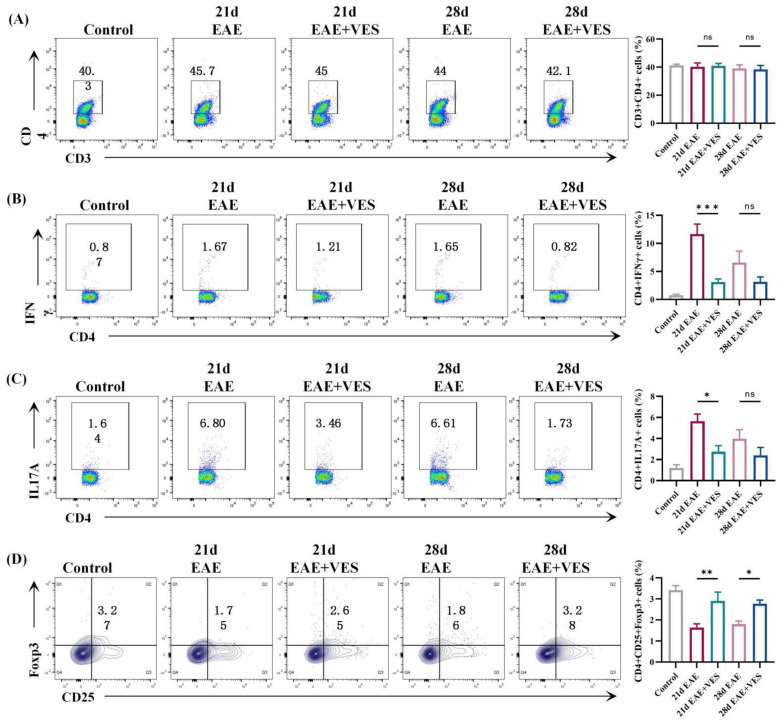
VES modulates peripheral blood T cell differentiation in EAE mice. (**A**) Proportion of CD3^+^CD4^+^ cells; (**B**) Proportion of CD4^+^IFN−γ^+^ cells; (**C**) Proportion of CD4^+^IL−17A^+^ cells; (**D**) Proportion of CD4^+^CD25^+^Foxp3^+^ cells (* *p* < 0.05, ** *p* < 0.01, *** *p* < 0.001; *n* = 5).

In peripheral blood (Figure 3), VES had no impact on overall CD4^+^ T cell numbers but significantly reduced Th1 (*p* < 0.001) and Th17 (*p* < 0.05) cell frequencies during the acute phase (day 21). This effect diminished during the remission phase (day 28), paralleling the spontaneous recovery observed in untreated mice. However, VES-induced Treg expansion persisted throughout both phases (*p* < 0.01 and *p* < 0.05, respectively).

These results suggest that VES rebalances peripheral T cell subsets in EAE by concurrently suppressing pathogenic Th1/Th17 responses and promoting Treg-mediated immunoregulation. This dual modulatory action supports its potential as a therapeutic agent for neuroinflammatory disorders such as multiple sclerosis.

### 2.4. VES Preserves BBB Integrity and Enhances Remyelination in EAE

**Figure 4:**
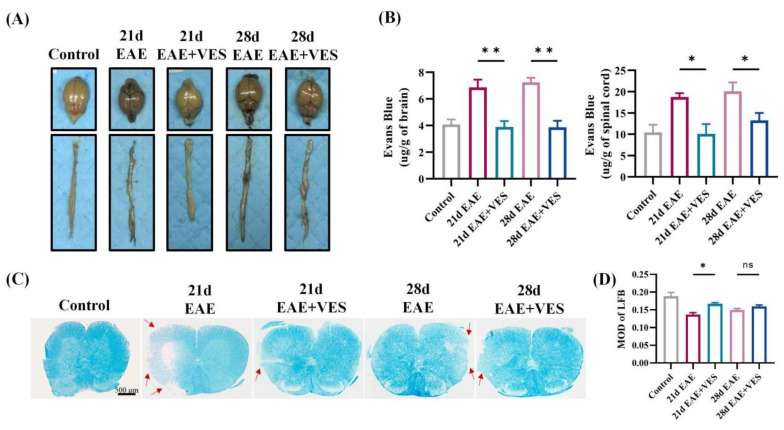
VES improves BBB integrity and reduces demyelination in EAE mice. (**A**) Representative images of brain and spinal cord tissues; (**B**) Quantification of Evans Blue extravasation; (**C**) LFB staining of spinal cord sections, arrows show the demyelination; (**D**) Quantification of LFB staining optical density (* *p* < 0.05, ** *p* < 0.01; *n* = 3).

BBB integrity was evaluated using Evans Blue (EB) extravasation. Control mice showed no dye infiltration, indicating intact BBB function. In contrast, EAE mice exhibited substantial EB accumulation in the brain and spinal cord at both acute (day 21) and chronic (day 28) phases, reflecting pronounced BBB disruption. VES treatment significantly reduced dye leakage (Figure 4A), with quantitative analysis confirming lower EB levels in both brain (*p* < 0.01) and spinal cord (*p* < 0.05) compared to untreated EAE mice (Figure 4B), indicating effective BBB protection and diminished inflammatory infiltration.

LFB staining was used to assess myelin pathology. Control mice displayed dense, intact myelin, whereas EAE mice showed marked demyelination by day 21, characterized by reduced staining and myelin fragmentation. VES significantly attenuated this demyelination (*p* < 0.05; Figure 4C,D). By day 28, spontaneous remyelination in the EAE group narrowed the difference between groups, suggesting that VES may accelerate myelin repair. Together, these results demonstrate that VES maintains BBB integrity, reduces neuroinflammation, and promotes remyelination, highlighting its neuroprotective efficacy in EAE.

### 2.5. VES Modulates Microglial Activation Status In Vivo and In Vitro

#### 2.5.1. VES Attenuates Microglial Overactivation and Promotes a Shift in Activation State in the Spinal Cord of EAE Mice

**Figure 5:**
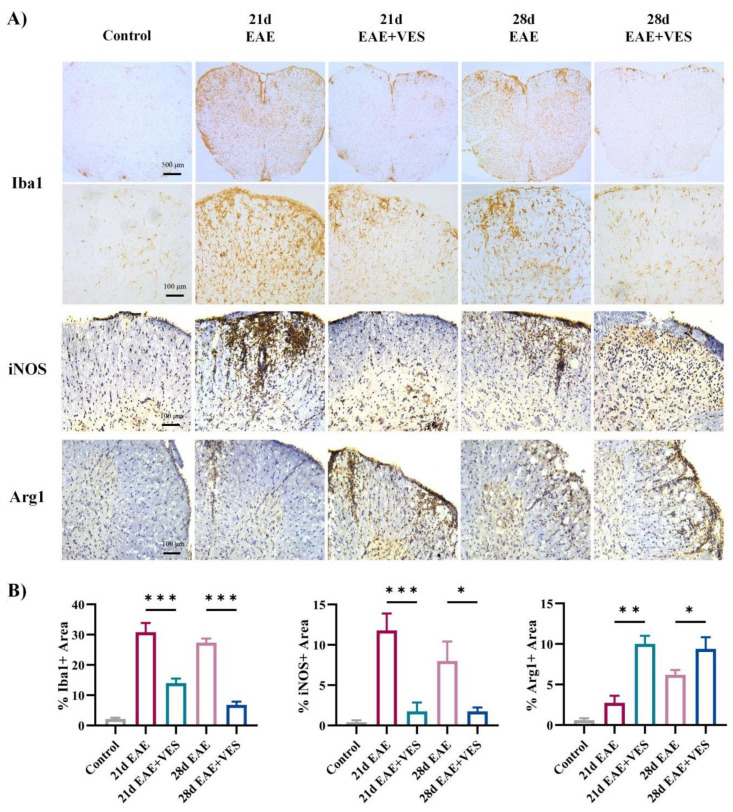
VES modulates microglial activation status in EAE spinal cord. (**A**) Immunohistochemical staining for Iba1, iNOS, and Arg1; (**B**) Quantification of Iba1^+^, iNOS^+^, and Arg1^+^ areas (* *p* < 0.05, ** *p* < 0.01, *** *p* < 0.001; *n* = 5).

Immunohistochemical analysis (Figure 5A) revealed that spinal cord microglia (Iba1^+^) in control mice displayed a resting morphology, with small cell bodies, extended processes, and faint Iba1 staining. In contrast, EAE mice (days 21 and 28) exhibited pronounced microglial activation, especially in the anterior horn white matter, characterized by enlarged somas, shortened processes, and intensified Iba1 labeling (*p* < 0.001). VES treatment significantly reduced microglial activation, restoring a quiescent phenotype and lowering Iba1^+^ cell density (*p* < 0.001). To gain initial insight into the microglial activation profile, we examined the expression of the canonical markers iNOS (often associated with pro-inflammatory responses) and Arg1 (often associated with immunoregulatory functions). Microglial analysis showed a strong upregulation of iNOS in EAE mice (*p* < 0.001), while Arg1 expression remained low. VES treatment markedly suppressed iNOS levels (*p* < 0.001, *p* < 0.05) and enhanced Arg1 expression (*p* < 0.01, *p* < 0.05; Figure 5B). These shifts in marker expression suggest that VES facilitates a change in microglial activation state, reducing a pro-inflammatory profile and promoting one associated with tissue repair.

#### 2.5.2. VES Directly Modulates Microglial Activation In Vitro

**Figure 6:**
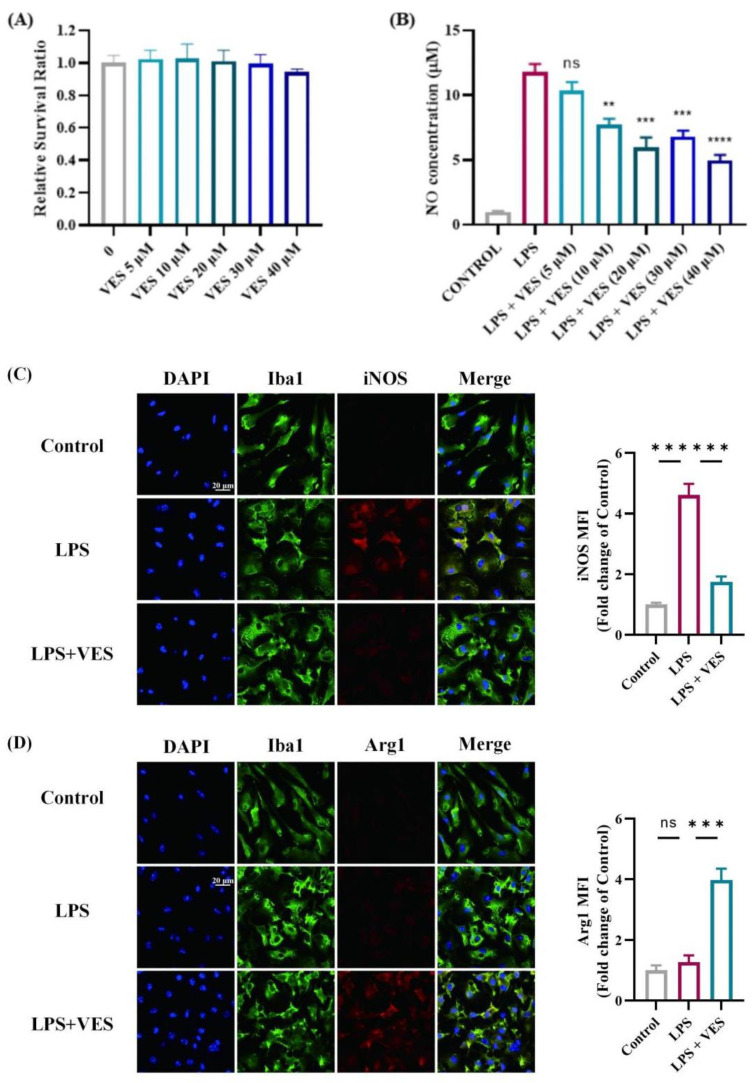
VES promotes microglial activation in vitro. (**A**) Cytotoxic effects of VES on primary microglia measured by CCK-8 assay; (**B**) determination of Optimal VES Concentration by NO Assay; (**C**) immunofluorescence staining of Iba1 and iNOS in microglia, with quantitative analysis of iNOS; (**D**) immunofluorescence staining of Iba1 and Arg1 in microglia, with quantitative analysis of Arg1 (** *p* < 0.01, *** *p* < 0.001, **** *p* < 0.0001; *n* = 5).

To exclude the influence of other immune cells, we assessed the direct effect of VES on microglia using a purified culture model (Figure 6). Prior to evaluating polarization, the cytotoxicity of VES was assessed using a CCK-8 assay. Microglia were treated with different concentrations of VES (5, 10, 20, 30, and 40 μM) for 24 h. No significant differences in cell viability were observed compared to the untreated control group, indicating that VES had no cytotoxic effect on microglial cells (Figure 6A). To determine the optimal intervention concentration, NO release was measured following LPS stimulation and subsequent VES treatment. VES at 20 μM significantly reduced NO production (*p* < 0.01), with no further improvement observed at higher concentrations (Figure 6B); thus, 20 μM was selected for subsequent experiments.

In control conditions, microglia displayed a resting morphology with elongated processes and minimal iNOS and Arg1 expression (Figure 6C,D). LPS stimulation induced a marked shift to an activated state, characterized by enlarged cell bodies, retracted processes, and strong iNOS expression (*p* < 0.001). Co-treatment with LPS and VES significantly suppressed iNOS levels while increasing the proportion of Arg1-positive microglia (*p* < 0.001). These in vitro findings corroborate the in vivo data and demonstrate that VES can directly influence microglial activation, suppressing a marker of pro-inflammatory activity and promoting one linked to alternative functions.

### 2.6. Proteomic Analysis of VES Modulation in the EAE Inflammatory Microenvironment

**Figure 7:**
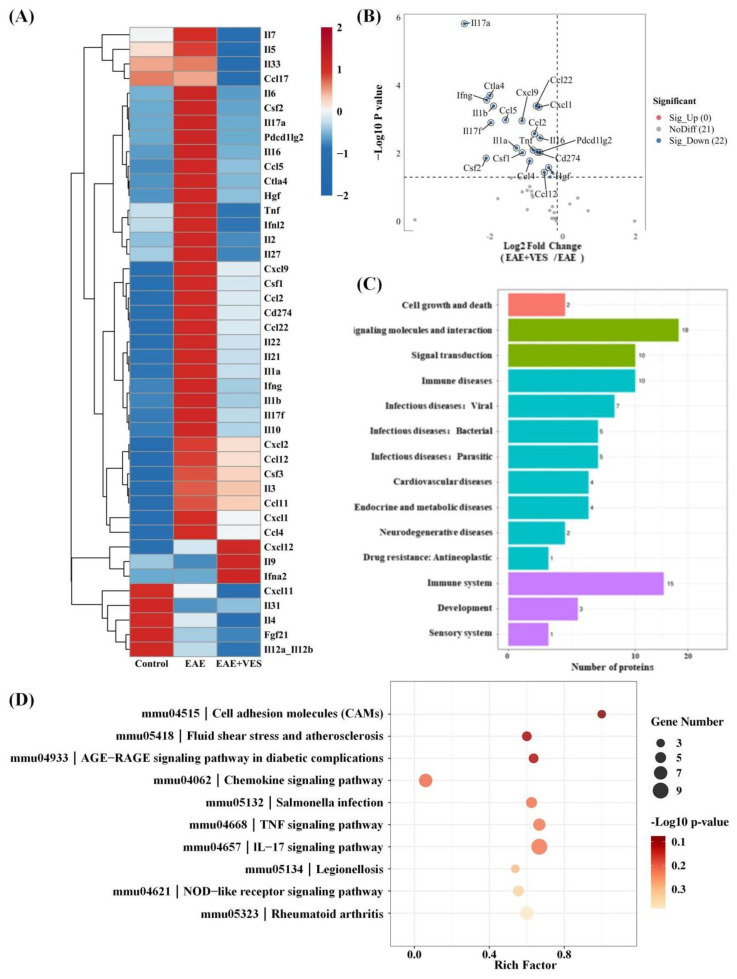
Differential protein expression in response to VES treatment. (**A**) Heatmap of protein expression; (**B**) volcano plot of differential expression; (**C**) KEGG functional classification; (**D**) KEGG pathway enrichment analysis (*n* = 4).

Proteomic analysis of spinal cord tissues from EAE and VES-treated (EAE + VES) mice using the OLINK platform identified 22 inflammation-associated proteins significantly downregulated by VES (Figure 7A,B). These included chemokines (e.g., CCL2, CXCL9) and interleukins (e.g., IL-1β, IL-6, IL-17a) involved in Th1/Th17 differentiation, immune cell migration, and M1 microglial activation. The pronounced downregulation of directly neurotoxic mediators, particularly TNF and IL-1β (Figure 7A), which are known to induce neuronal apoptosis and excitotoxicity, strongly suggests a mechanism for enhanced neuronal survival. Furthermore, the suppression of key signaling pathways, including the TNF and IL-17 signaling pathways (Figure 7B), is directly relevant to neuro-glial crosstalk. IL-17a potently drives astrocytic activation towards a detrimental A1 state, while TNF disrupts synaptic integrity and oligodendrocyte function. By attenuating these pathways, VES likely facilitates a shift in the glial phenotype, fostering an environment that supports protective astrocyte–microglia–neuron interactions essential for efficient brain functioning. This is corroborated by the downregulation of Csf1 (M-CSF), a master regulator of microglial proliferation and activation, indicating a transition towards a more homeostatic glial state. These results suggest that VES mitigates CNS inflammation by reducing pro-inflammatory cytokine release, thereby limiting T cell infiltration and suppressing microglial activation, ultimately creating a microenvironment that is conducive to neuronal preservation, functional recovery, and restorative neuro-glial communication.

Kyoto encyclopedia of genes and genomes (KEGG) pathway analysis revealed significant enrichment in inflammatory and immune signaling pathways, including TNF, chemokine, IL-17, and rheumatoid arthritis pathways (Figure 7C). Notably, differentially expressed proteins were also enriched in the mmu4518 pathway (fluid shear stress and atherosclerosis), which is regulated by Nrf2—a key transcription factor that counteracts oxidative stress, inhibits pro-inflammatory mediators (e.g., TNF-α, IFN-γ), and preserves vascular stability. This mechanism parallels that of dimethyl fumarate (DMF), a clinically approved Nrf2 activator used in multiple sclerosis, indicating that VES may exert neuroprotective effects through a similar Nrf2-dependent pathway.

Further enrichment in the NOD-like receptor and cell adhesion molecule pathways (mmu4621), mmu4515 suggests that VES may also modulate innate immunity and reinforce blood–brain barrier integrity. Together, these findings indicate that VES targets multiple inflammatory pathways, reshaping the CNS immune microenvironment and offering molecular support for its therapeutic potential in neuroinflammatory disorders.

### 2.7. VES Confers Neuroprotection via Nrf2 Pathway Activation

To elucidate the molecular mechanism underlying VES-mediated neuroprotection in EAE, we investigated the activation of the Nrf2 signaling pathway both in vivo and in vitro.

#### 2.7.1. In Vivo Activation of Nrf2 by VES

**Figure 8:**
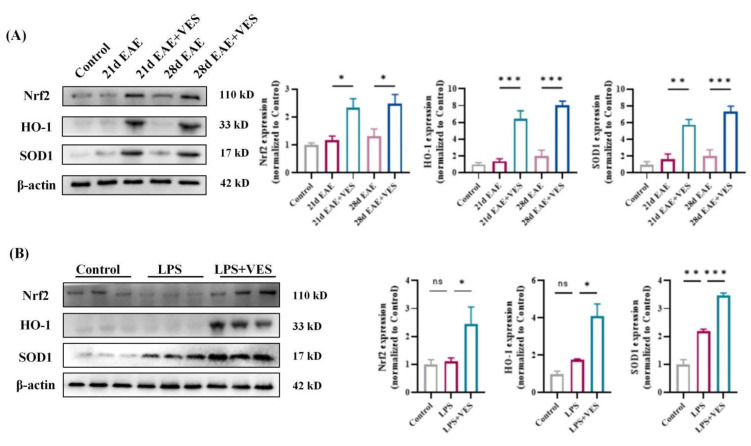
VES activates the Nrf2 signaling pathway both in vivo and in vitro. (**A**) Protein expression levels of Nrf2, HO-1, SOD1, and β-actin in mouse spinal cord; (**B**) protein expression levels of Nrf2, HO-1, SOD1, and β-actin in microglia (* *p* < 0.05, ** *p* < 0.01, *** *p* < 0.001; *n* = 4).

In EAE mice, spinal cord expression of Nrf2 and its downstream antioxidant proteins HO-1 and SOD1 showed a slight, non-significant increase from day 21 to day 28 compared to controls, indicating a limited endogenous response to oxidative stress. In contrast, VES treatment markedly enhanced Nrf2 activity and significantly upregulated HO-1 (*p* < 0.05) and SOD1 (*p* < 0.001) levels (Figure 8A). These results suggest that VES activates the Nrf2 pathway beyond the baseline compensatory response, promoting antioxidant and anti-inflammatory defense mechanisms to mitigate neurodegeneration.

#### 2.7.2. In Vitro Confirmation in Microglia

In LPS-stimulated microglia, SOD1 expression increased significantly (*p* < 0.01), while Nrf2 and HO-1 levels rose only modestly, reflecting a restrained cellular response to inflammatory stress. VES co-treatment significantly promoted Nrf2 nuclear translocation and further elevated HO-1 (*p* < 0.05) and SOD1 (*p* < 0.001) expression (Figure 8B). These in vitro findings align with in vivo results, demonstrating that VES directly activates the Nrf2/HO-1/SOD1 axis in microglia to suppress inflammation and modulate polarization. Collectively, these data establish Nrf2 pathway activation as a central mechanism by which VES exerts its anti-inflammatory and neuroprotective effects in EAE.

## 3. Discussion

This study comprehensively evaluated the therapeutic efficacy and molecular mechanisms of VES in a mouse model of EAE. The results show that VES markedly alleviates both clinical symptoms and neuropathological changes in EAE through a multi-faceted mechanism involving peripheral immune regulation and CNS protection.

VES exhibited potent immunomodulatory activity by selectively inhibiting the differentiation of pro-inflammatory Th1 and Th17 cells while enhancing the expansion of regulatory T cells (Tregs). These findings are consistent with earlier reports on immune modulation in multiple sclerosis [25,26,27,28]. Importantly, the immunoregulatory effects of VES followed a time-dependent trajectory: suppression was apparent but not statistically significant during the acute phase (day 21), whereas significant modulation was observed in the chronic phase (day 28). This temporal pattern suggests a progressive regulatory effect, resembling the dynamics of chronic neuroinflammation described by Goverman et al. [29], and highlights the potential of VES for long-term treatment of neuroinflammatory conditions.

VES also provided central nervous system protection. It significantly preserved BBB integrity, as demonstrated by reduced Evans blue dye leakage compared to untreated EAE controls. This finding supports previous evidence linking BBB disruption to neurodegeneration [30,31,32]. By maintaining BBB integrity, VES may prevent peripheral immune cell infiltration into the CNS and promote a reparative environment, consistent with the mechanisms proposed by Ortiz et al. [33]. Additionally, VES protected against demyelination. LFB staining revealed reduced myelin loss during the acute phase in VES-treated mice. Although partial spontaneous remyelination occurred in controls by day 28, the VES group showed markedly greater recovery. These results suggest that VES enhances myelin repair, likely by facilitating the differentiation and maturation of oligodendrocyte precursor cells (OPCs), in line with the remyelination mechanisms described by Franklin et al. [34] and Fancy et al. [35].

Our in vivo and in vitro studies consistently showed that VES attenuates microglial overactivation and shifts their phenotype from pro-inflammatory M1 to neuroprotective M2. Immunohistochemical analysis in EAE mice showed that VES reduced Iba1^+^ microglial density and restored a resting morphology, accompanied by downregulation of iNOS and upregulation of Arg1, indicating a shift toward M2 polarization. In vitro assays further confirmed that VES acts directly on microglia to suppress NO and iNOS expression and enhance Arg1 expression in the absence of other immune cells, indicating a cell-autonomous effect. This shift is clinically significant, as dysregulated microglial activation is a central feature of neuroinflammatory diseases such as multiple sclerosis [14,36,37]. In the pathogenesis of MS and its animal model EAE, M1-polarized microglia promote demyelination and axonal damage through the release of pro-inflammatory cytokines and reactive nitrogen species [38]. Promoting M2 polarization has been shown to facilitate tissue repair and neuroregeneration [39,40,41]. Importantly, our data showed that VES at a concentration of 20 μM was sufficient to suppress LPS-induced NO release without inducing cytotoxicity, further supporting its therapeutic potential. This finding is consistent with reports that vitamin E derivatives can modulate redox balance and inflammatory signaling in microglia through inhibition of NF-κB and activation of Nrf2 pathways [42,43]. These signaling cascades are critical regulators of microglial phenotype and could underlie the effects observed in our study. Together, these results not only support VES as a promising immunomodulatory agent for neuroinflammatory disorders but also emphasize the broader therapeutic value of targeting microglial plasticity. Future studies may explore the molecular pathways mediating VES-induced polarization, such as TLR4-NF-κB, JAK/STAT, or PI3K/Akt signaling axes.

At the molecular level, this study is the first to demonstrate that VES exerts neuroprotective effects by activating the Nrf2/HO-1 signaling pathway. Proteomic analysis revealed significant upregulation of Nrf2-related proteins following VES treatment, which was further validated by Western blot showing increased expression of Nrf2 and its downstream targets HO-1 and SOD1. As a key regulator of oxidative stress and neuroinflammation, the Nrf2 pathway represents a promising therapeutic target [44,45,46,47]. Supporting our results, Li et al. [48] reported that Nrf2 activation drives microglia toward an anti-inflammatory phenotype. VES-induced Nrf2 activation was consistently observed in both in vivo and in vitro models, confirming its mechanistic relevance. Compared to conventional Nrf2 activators such as dimethyl fumarate, VES offers broader effects by simultaneously enhancing Nrf2 signaling, modulating microglial polarization, and influencing peripheral immune balance. This multifaceted activity aligns with the multi-target neuroprotective strategies proposed by Farjam et al. [49]. Moreover, Li et al. [48] linked Nrf2-driven microglial shifts to inflammation resolution, further underscoring the clinical significance of our findings.

Therapeutically, the multi-target profile of VES provides a significant advantage in addressing neuroinflammatory diseases. Unlike traditional single-target agents, VES simultaneously modulates key pathological processes, including: (1) rebalancing peripheral immunity, (2) preserving BBB integrity, (3) directing microglial polarization, and (4) activating endogenous antioxidant defenses. This integrated approach may be particularly well-suited for complex disorders like MS, which involve interconnected pathological mechanisms [50]. Recent studies increasingly support multi-target strategies in the treatment of neurodegenerative diseases.

Nonetheless, this study has limitations. First, the molecular mechanisms by which VES activates the Nrf2 pathway remain unclear; while proteomic data suggest potential signaling routes, further validation is needed. Second, the pathways through which VES promotes myelin regeneration require deeper investigation. Additionally, the relatively small sample sizes in certain experimental groups (e.g., flow cytometry, proteomics) may limit the statistical power and generalizability of some conclusions. Future studies with larger cohorts are warranted to confirm and extend these findings. Furthermore, this study was conducted exclusively in male mice. Given the known sexual dimorphism in MS incidence and EAE susceptibility, future studies in female animals and direct comparisons between sexes are essential to fully characterize the therapeutic potential of VES across populations. Finally, although the EAE model is widely used in MS research, it does not fully capture the complexity of the human disease [51].

## 4. Materials and Methods

### 4.1. Animals

Adult male C57BL/6J mice (8 weeks old; *n* = 100) and neonatal males (postnatal days 0–3; *n* = 6), all of specific pathogen-free (SPF) grade, were procured from Beijing Vital River Laboratory Animal Technology Co., Ltd. (Beijing, China). Animals were maintained at the Laboratory Animal Center of Shenzhen University under standardized conditions of temperature and humidity, with a 12 h light/dark cycle. Food and water were supplied ad libitum. All procedures were reviewed and approved by the Institutional Animal Care and Use Committee (IACUC) of Shenzhen University (Approval No. IACUC-202400090, 3 June 2024) and carried out in accordance with national guidelines for the ethical use of laboratory animals in China.

### 4.2. Induction of EAE

EAE was induced on day 0 via subcutaneous immunization with an emulsion containing 250 μg of myelin oligodendrocyte glycoprotein peptide (MOG_35-55_; MCE, Princeton, NJ, USA) and complete Freund’s adjuvant (CFA; Chondrex, Woodinville, WA, USA), supplemented with 5 mg/mL heat-killed Mycobacterium tuberculosis H37RA, resulting in a final MOG concentration of 2 mg/mL [52,53].To prepare the emulsion, MOG_35-55_ was first dissolved in sterile PBS at an initial concentration of 1.25 mg/mL and mixed with an equal volume of 5 mg/mL CFA. The mixture was emulsified thoroughly on ice using a high-speed homogenizer to form a stable water-in-oil emulsion. Each mouse was anesthetized with isoflurane and subcutaneously injected with 100 μL of the emulsion at two sites on the flanks near the base of the limbs, for a total volume of approximately 400 μL per mouse. Pertussis toxin (PTX; List Biological Laboratories, Campbell, CA, USA) was reconstituted in PBS to prepare a 100 μg/mL stock solution and stored at 4 °C. Immediately before use, it was diluted in PBS to a working concentration of 2 μg/mL. Mice received intraperitoneal injections of 200 μL PTX working solution on days 0 and 2.

Clinical signs were monitored daily and scored as follows: 0, no symptoms; 1, limp tail or mild hind limb weakness; 2, limp tail and hind limb weakness; 3, partial hind limb paralysis; 4, complete hind limb paralysis; 5, moribund. Mice reaching a score of 5 were humanely euthanized. Intermediate scores were assigned in 0.5 increments [54].

### 4.3. Grouping and Treatment

Following immunization, mice were randomly assigned to either EAE or EAE + VES groups, with non-immunized mice designated as the Control group. The experimental design comprised five groups: (1) Control, (2) 21-day EAE, (3) 21-day EAE + VES, (4) 28-day EAE, and (5) 28-day EAE + VES. The 21-day time point was selected to model the acute phase of EAE, corresponding to peak neuroinflammation, while the 28-day time point was used to evaluate chronic pathology and potential recovery. From days 14 to 20 post-immunization, VES (3 mg/kg) was administered daily via intraperitoneal injection to the EAE + VES groups. VES was dissolved in a vehicle containing 3% DMSO, 40% PEG 300, 5% Tween 100, and 52% saline. Equivalent volumes of vehicle were administered to the Control and EAE groups. Mice in the 21-day groups were euthanized immediately after the treatment period, whereas those in the 28-day groups were maintained until day 28 to assess prolonged effects. The VES dosage was based on previous studies demonstrating its anti-inflammatory efficacy.

### 4.4. Flow Cytometry

Peripheral blood and spleens were collected from isoflurane-anesthetized mice. Approximately 200 μL of peripheral blood was obtained via retro-orbital puncture into heparinized tubes. Mice were subsequently euthanized by overdose anesthesia, and spleens were dissected on ice and transferred into pre-cooled PBS.

Peripheral blood was treated with 1 mL red blood cell lysis buffer at room temperature for 10 min. Following lysis, samples were centrifuged at 500× *g* for 5 min at 4 °C. The pellet was washed once with PBS and resuspended in 1 mL RPMI 1640 medium. Spleens were mechanically dissociated through a 70 μm cell strainer using pre-cooled PBS, and the filtrate was adjusted to a final volume of 10 mL. After centrifugation (500× *g*, 5 min, 4 °C), red blood cells were lysed using 2 mL red blood cell lysis buffer for 10 min at room temperature. Cells were subsequently washed with PBS, centrifuged, and resuspended in RPMI 1640 medium. Cell counts were determined using a hemocytometer, and 5 × 10^6^ cells were used per sample. To assess intracellular IFN-γ and IL-17A production, single-cell suspensions were stimulated for 5 h at 37 °C in a 5% CO_2_ incubator using Leukocyte Activation Cocktail (BD Biosciences, Milpitas, CA, USA) diluted 1:500 in RPMI 1640 (freshly prepared).

Cells were first incubated with fluorophore-conjugated surface antibodies (FITC-conjugated anti-CD3, FITC- or BV510-conjugated anti-CD4, and APC-conjugated anti-CD25; BD Biosciences) in 100 μL antibody diluent (5 μg/mL in pre-cooled PBS) at 4 °C for 40 min in the dark. After washing with PBS and centrifugation (500× *g*, 5 min, 4 °C), cells were fixed with 1 mL Fixation and Permeabilization Solution (BD Biosciences) for 40 min at 4 °C in the dark. Cells were then washed and permeabilized with 1 mL 1× Perm/Washing Working Solution for 10 min. Intracellular staining was performed using PE-conjugated anti-Foxp3, PE-conjugated anti-IFN-γ, and BV421-conjugated anti-IL-17A antibodies (BD Biosciences) diluted in 1× Perm/Washing Working Solution at 5 μg/mL, followed by incubation at 4 °C for 40 min in the dark. After final washes, cells were resuspended in 200 μL PBS, filtered through a 200-mesh nylon strainer, and analyzed on a fluorescence-activated cell sorter (FACS, Edwardsville, IL, USA).

### 4.5. Evans Blue Assay for Blood–Brain Barrier (BBB) Permeability

A 2% (*w*/*v*) Evans blue solution was administered intravenously via the tail vein at 4 mL/kg. After 2 h, mice were perfused with phosphate-buffered saline (PBS), and the brain and spinal cord were collected, photographed, and weighed. Tissues were incubated overnight in 1 mL formamide at 56 °C. The following day, samples were centrifuged at 10,000× *g* for 10 min, and supernatants were collected for absorbance measurement at 635 nm. Evans blue concentrations were calculated from a standard curve and normalized to tissue weight.

### 4.6. Luxol Fast Blue (LFB) Staining and Immunohistochemistry

Mice were anesthetized with intraperitoneal pentobarbital sodium (200 μL) and perfused transcardially with 50 mL cold PBS followed by 50 mL cold 4% paraformaldehyde (PFA, Hillside, NJ, USA). Lumbar spinal cords were dissected, post-fixed, dehydrated, and cryosectioned at 20 μm thickness.

For LFB staining, sections were incubated overnight in LFB solution (Solarbio, Beijing, China) at room temperature, differentiated in 95% ethanol for 30 s, rinsed in distilled water, treated with Luxol differentiation solution for 15 s, followed by 70% ethanol for 30 s, and rinsed again. Sections were then dehydrated through graded ethanol, cleared in xylene, and mounted with neutral balsam.

For immunohistochemistry, sections were blocked and permeabilized with 5% goat serum containing 0.3% Triton X-100 for 1 h at room temperature, then incubated overnight at 4 °C with primary antibodies against Iba-1 (1:300), iNOS (1:200), and Arg1 (1:200) (all from CST, San Antonio, TX, USA). Following PBS washes, sections were incubated with HRP-conjugated anti-rabbit IgG secondary antibody (1:200; CST) for 1 h at room temperature and developed with DAB for 2–5 min. Sections were dehydrated, cleared, and mounted as described above. Images were acquired and analyzed using ImageJ 1.54p software Graphpad Prism 9.5.

### 4.7. Olink Proteomics

Lumbar spinal cords were freshly isolated from euthanized mice, washed thoroughly in PBS to remove residual blood, snap-frozen in liquid nitrogen, and shipped on dry ice to BGI Genomics (Shenzhen, China) for proteomic profiling. Cytokine expression was quantified using the Olink^®^ Target 48 Mouse Cytokine panel (Uppsala, Sweden), which targets 48 inflammatory cytokines (listed in Table 1), employing Proximity Extension Assay (PEA) technology for high-specificity, high-sensitivity quantification.

Each cytokine is detected by a matched pair of oligonucleotide-labeled antibodies that bind distinct epitopes of the target protein. When both antibodies bind to the same protein, their attached DNA probes are brought into proximity, allowing for hybridization and enzymatic extension to form amplifiable double-stranded DNA. This highly specific reaction ensures low background and minimal false positives, even for low-abundance proteins. The resulting DNA is quantified using real-time qPCR, and protein abundance is inferred from Cq values.

To ensure experimental reliability and reproducibility, quality control (QC) procedures were rigorously followed. Each sample included three internal controls—incubation, extension, and detection controls—to monitor assay performance. In addition, each assay plate included three external controls (negative control, sample control, and calibrator), analyzed in technical duplicates or triplicates as appropriate. Internal and external QC criteria required a standard deviation <0.2 for within-sample internal controls and <0.5 for external controls. Individual sample QC was assessed based on the deviation of incubation and detection control NPX values from the plate median, with values ≤0.3 considered acceptable; samples exceeding this threshold were flagged for QC caution.

### 4.8. Primary Microglial Cell Isolation and Culture

Reagents and materials: Poly-D-lysine (PDL, 10 mg; Sigma-Aldrich, St. Louis, MO, USA) was dissolved in 1 mL Dulbecco’s phosphate-buffered saline (DPBS, Gibco) to prepare a 100× stock solution (10 mg/mL), aliquoted, and stored at −20 °C. Working solution (1×, 100 µg/mL) was freshly prepared by dilution with DPBS before use. Culture flasks or plates were precoated with 1× PDL overnight at 4 °C. The next day, excess PDL was removed, and the flasks were rinsed three times with DPBS, air-dried in a biosafety cabinet, and UV-sterilized for 1 h prior to use. Complete culture medium was prepared by mixing DMEM (Gibco), 10% fetal bovine serum (FBS, Gibco), and 1% penicillin–streptomycin (P/S, Gibco). Dissection medium (DM) consisted of Hank’s Balanced Salt Solution (HBSS):HEPES:P/S in a 90:10:1 ratio.

Microglial cell isolation: Primary microglia were obtained from postnatal day 0 to 3 (P0–P3) SPF-grade neonatal mice. Following euthanasia and 75% ethanol disinfection, the cerebral cortices were rapidly dissected under a stereomicroscope and transferred into pre-chilled DM in a 6 cm culture dish. The cortical tissue was minced into small pieces, transferred into a 50 mL conical tube, and adjusted to 30 mL with DM. Tissue was enzymatically dissociated using 0.25% trypsin and DNase I at 37 °C for 15 min. Digestion was terminated by adding 5 mL of complete medium. Cells were centrifuged at 400× *g* for 5 min, resuspended in 5 mL complete medium, and filtered through a 70 μm cell strainer to remove undigested fragments. After a second centrifugation step (400× *g*, 5 min), cells were resuspended in complete medium and plated into PDL-coated T75 flasks. Cultures were maintained at 37 °C in a humidified incubator with 5% CO_2_.

Microglial cell purification: On day 2, the medium was replaced after gentle rinsing with DPBS to remove debris and unattached cells. Medium was refreshed again on days 5 and 8. After approximately 12–14 days, a dense confluent glial layer formed, composed primarily of astrocytes at the bottom, with microglia and a small number of oligodendrocytes growing on top. To isolate microglia, flasks were placed on a rotary shaker at 37 °C and shaken at 200 rpm for 2 h. The supernatant containing detached microglia and oligodendrocytes was collected and transferred to PDL-coated plates. After 2 h of incubation, loosely attached cells were removed by tapping the plate gently and replacing the medium. Due to their weak adhesion, oligodendrocytes were mostly eliminated during this step, resulting in a population highly enriched for microglial for downstream experiments.

### 4.9. In Vitro Neuroinflammation Model Induction

To establish an in vitro model of neuroinflammation, primary microglial cells were stimulated with lipopolysaccharide (LPS, Sigma-Aldrich), a well-characterized pathogen-associated molecular pattern (PAMP). Based on previously published studies [55,56,57], LPS was applied at a final concentration of 100 ng/mL. Microglia were randomly assigned to three experimental groups: a control group (no treatment for 24 h), an LPS group (treated with LPS for 24 h), and an LPS + VES group (treated with LPS for 12 h, followed by addition of VES for another 12 h).

### 4.10. Cell Viability Assay (CCK-8)

To evaluate the potential cytotoxicity of VES and ensure that its anti-inflammatory effects were not confounded by reduced viability, a Cell Counting Kit-8 (CCK-8, Dojindo, Rockville, MD, USA) assay was performed. Purified microglia were seeded into 96-well plates and treated with VES at concentrations of 5, 10, 20, 30, and 40 μM for 24 h. An untreated control group and a blank (medium-only) group were included. After treatment, 10 μL of CCK-8 reagent was added to each well and incubated at 37 °C for 1.5 h. Absorbance at 450 nm was measured using a microplate reader (BioTek, Ahmedabad, Indian), and cell viability was calculated relative to the control group.

### 4.11. Nitric Oxide (NO) Production Assay

To determine the optimal VES concentration for anti-inflammatory efficacy, microglia were stimulated with LPS (100 ng/mL) for 24 h, followed by VES treatment at different concentrations (5, 10, 20, 30, 40 μM) for an additional 24 h. The NO concentration in the culture supernatant was determined using the Griess reagent. Reaction mixtures were prepared in a 96-well plate. Absorbance was measured at 540 nm, and NO levels were calculated based on a standard curve generated using known concentrations of sodium nitrite.

### 4.12. Immunofluorescence

Cells cultured on coverslips were fixed with 4% paraformaldehyde for 20 min, permeabilized with 0.3% Triton X-100 for 15 min, and blocked in 5% goat serum for 1 h at room temperature. Primary antibodies against Iba-1 (1:300), iNOS (1:200), and Arg1 (1:200) (CST) were applied overnight at 4 °C. Following PBS washes, Alexa Fluor 488- or 594-conjugated secondary antibodies (1:200; CST) were added for 1 h in the dark. Nuclei were counterstained with DAPI, and coverslips were mounted using antifade medium. Images were acquired by fluorescence microscopy and analyzed with ImageJ.

### 4.13. Western Blotting

Lysates from spinal cord and microglial samples were prepared using RIPA buffer containing PMSF and phosphatase inhibitors. Protein concentrations were determined by BCA assay. Equal amounts of protein were separated via SDS-PAGE (4–20%) and transferred to PVDF membranes. Membranes were blocked and incubated overnight at 4 °C with primary antibodies against β-actin (1:10,000; CST), Nrf2 (1:1000; CST), HO-1 (1:2000; Abcam), and SOD1 (1:2000; Abcam). After washing, membranes were incubated with HRP-conjugated anti-rabbit IgG (1:1000; CST) for 1 h at room temperature. Bands were visualized using an enhanced chemiluminescence system (Thermo Scientific, Waltham, MA, USA) and quantified with ImageJ.

### 4.14. Statistical Analysis

All data were analyzed using GraphPad Prism 9.5.0 and are presented as mean ± SEM. Statistical comparisons were performed using one-way ANOVA followed by Tukey’s post hoc test. Significance was considered at *p* ≤ 0.05 (* *p* < 0.05, ** *p* < 0.01, *** *p* < 0.001).

## 5. Conclusions

In summary, this study establishes the therapeutic potential of VES in the EAE model and elucidates its underlying mechanisms. VES provides neuroprotection by modulating peripheral immunity, preserving blood–brain barrier integrity, regulating microglial polarization, and activating the Nrf2 pathway. These results deepen our understanding of neuroinflammatory processes and offer a strong preclinical foundation for developing VES as a treatment for multiple sclerosis. Future research should focus on: (1) identifying upstream regulators of Nrf2 activation by VES; (2) uncovering the cellular mechanisms of VES-induced myelin repair; and (3) evaluating its efficacy in primate models to facilitate clinical translation.

## Figures and Tables

**Table 1:** Cytokines included in the Olink^®^ Target 48 Mouse Cytokine panel.

Cytokines	Cytokines	Cytokines	Cytokines
IL1a	IL12	Cxcl11	Ccl17
ILIb	IL16	Cxcl12	Fg121
IL2	ILI7a	Ccl22	Cila4
IL3	ILI7f	Csl1	Cd274
IL4	IL21	Csl2	Tnf
IL5	IL27	Csl3	IFNa2
IL6	IL31	Ccl2	IFNg
IL7	IL33	Ccl4	IFNl2
IL9	Cxcl1	Ccl5	Hgf
II10	Cxcl2	Ccl11	Pdcd1lg2
ILI2a, ILI2b	Cxcl9	Ccl12	

## Data Availability

The datasets used and/or analyzed during the current study will be available from the corresponding author on reasonable request.
